# Vitreous levels of pigment epithelium-derived factor and vascular endothelial growth factor in diabetic and non-diabetic retinopathy: associated factors and anatomical correlation

**DOI:** 10.1186/s40942-024-00556-2

**Published:** 2024-05-21

**Authors:** Rami Al-Dwairi, Tamam El-Elimat, Abdelwahab Aleshawi, Ahmed Al Sharie, Seren Al Beiruti, Abdallah K. Sharayah, Mohammed Allouh

**Affiliations:** 1 Division of Ophthalmology, Department of Special Surgery, Faculty of Medicine, Jordan University of Science and Technology, P. O. Box: 3030, Irbid, 22110 Jordan; 2 Department of Medicinal Chemistry and Pharmacognosy, Faculty of Pharmacy, Jordan University of Science and Technology, Irbid, 22110 Jordan; 3 Department of Pathology and Microbiology, Faculty of Medicine, Jordan University of Science and Technology, Irbid, 22110 Jordan; 4 Department of Anatomy, College of Medicine and Health Sciences, United Arab Emirates University, Al Ain, 15551 United Arab Emirates

**Keywords:** VEGF, Rhegmatogenous retinal detachment, PEDF, ELISA, Diabetic retinopathy

## Abstract

**Background:**

This study aims to investigate the factors affecting the vitreous levels of pigment epithelium-derived factor (PEDF) and vascular endothelial growth factor (VGEF) among patients with pars plana vitrectomy (PPV). Also, this study correlates the levels of PEDF with RRD characteristics.

**Methods:**

All patients who were scheduled for PPV for any indication were included in the study. They were divided into a case group which included patients with advanced PDR and a control group which included the remaining diagnoses. During the PPV, an undiluted vitreous sample was taken and the enzyme-linked immunosorbent assay method was utilized to measure the levels of VEGF and PEDF.

**Results:**

Eighty eyes were involved. Patients diagnosed with advanced PDR and endophthalmitis exhibited higher levels of VEGF. PEDF was affected inversely by the age of the patients and PEDF levels were higher in RRD and endophthalmitis cases. In patients with RRD, the level of PEDF was higher if the tear was found inferiorly, if the macula was detached, and with a longer duration of RRD.

**Conclusions:**

This study highlights the clinical importance of those biomarkers. Anti-VEGF-based treatment is the mainstay against PDR. PEDF may show a promising predictive values regarding patients with RRD.

## Background

Developing diabetic retinopathy (DR) is not universal in every patient with diabetic mellitus (DM). Duration of DM and poor glycemic control have essential function in the development and progression of DR. According to the severity, DR is categorized as non-proliferative DR (NPDR) or proliferative DR (PDR). Diabetic macular edema can be focal or diffuse, can be ischemic or non-ischemic, and can accompany any stage of the DR [1, 2]. Molecular biomarkers are used in DR to diagnose, predict, and follow up treatment responses. Examples of biomarkers include glycated hemoglobin A1c (HBA1c), vascular endothelial growth factor (VGEF), and pigment epithelium-derived factor (PEDF) [3].

The PEDF has multiple biological function with neuroprotective, antioxidative, and antiangiogenic effects. It belongs to the serine protease inhibitor family. It was first identified in fetal retinal pigment epithelium (RPE) cells as a neuro-differentiation factor [4, 5]. PEDF has a wide multi-organ effect, on the the retina, kidney, heart, liver, lungs, and brain. In these tissue, PEDF exhibits an essential role in maintaining and regulating microvascular homeostasis and the fluctuation in its level plays the task of organ’s vascular growth, development, and pathophysiology [5, 6]. For example, in the eye, deficiency of PEDF severely affects the capillary plexus density [7, 8]. The neuroprotective effect of PEDF plays a role in the protection of the retina in cases of rhegmatogenous retinal detachment (RRD) [9].

VEGF is a cytokine glycoprotein and it is a potent angiotnetic factor that mediate angiogenesis, endothelial wall growth, and vascular permeability. It is produced from capillary endothelial cells, RPE, ganglion cells, Müller cells, and astrocytes [10–14]. Increased levels of serum and VEGF are associated with the pathogenesis of DR [15]. For this, intravitreal anti-VEGF injections are currently one of the first line of treatment of patients with DR [16].

In this study, we examined the factors that affect the levels of PEDF and VEGF in patients who underwent pars plana vitrectomy (PPV) among different indications, especially patients with advanced PDR. In addition, this study investigates the correlation between PEDF levels and the clinical and anatomical characteristics of RRD.

## Methods

### Patients and data

This research was carried out at King Abdullah University Hospital (KAUH) between December 2020 and June 2022. KAUH is a university hospital affiliated with Jordan University of Science and Technology (JUST), and it is a tertiary referral center for surgical vitreoretinal cases in the North of Jordan. The study was approved by the IRB committee at KAUH (Approval No. 58/137/2021). Patients who had been scheduled to undergo pars plana vitrectomy (PPV) for any reason were asked to obtain a vitreous sample during the vitrectomy. The vitreous samples were tested for vascular endothelial growth factor (VEGF) and pigment epithelium-derived factor (PEDF). All patients gave written informed consent, and the study was conducted according to the guidelines of the Declaration of Helsinki and its subsequent amendments. The patients’ age, sex, past ophthalmic history, previous medical history, and co-morbidities were recorded. Additionally, the indication for PPV, the type of tamponade and the associated surgical procedures were documented. Lastly, the visual outcomes were measured.

The included participants were those patients who had been scheduled to undergo PPV. The exclusion criteria included patients who had previously undergone vitrectomy, patients with occlusion of the retinal veins, those who had less than two months duration of vitreous hemorrhage (VH), cases where obtaining a vitreous sample can result in a complication, patients who had received prior chemotherapy, and those who had previously undergone complicated cataract surgery where the fluid could dilute the vitreous.

Similar to our previous study, the participants were divided into two groups; cases vs. control [17]. The case group included patients with persistent VH and/or fibrovascular membranes (FVM) with tractional retinal detachment (TRD) (advanced proliferative diabetic retinopathy (PDR)). The modified Airlie house classifications was used for the grading [18, 19].

The control group comprised patients with vitreomacular interface diseases (i.e., vitreomacular traction (VMT), macular hole (MH), and non-diabetic epiretinal membranes (ERM)), rhegmatogenous retinal detachment (RRD), endophthalmitis, and drop of crystalline lens materials and/or drop of intraocular lens implants after complicated cataract surgery.

Intravitreal anti-VEGF injections were used in most patients in the first group preoperatively. They were analyzed and correlated with the level of the biomarkers according to the time and the number of injections given in the case group. Either aflibercept or ranibizumab were utilized in this study. In addition, the effect of panretinal photocoagulation laser sessions was studied. The possible effect of the associated cataract surgery in some patients during PPV was also studied. Moreover, the type of retinal tamponade after the PPV was collected, which included silicone oil, gas (SF6), and air.

A special analysis was performed for RRD cases alone. Certain characteristic of RRD was investigated and related to PEDF level including macula status (either the macula was attached or not), the location of the tear (superiorly or inferiorly), and the duration of symptoms of RRD. Using Snellen decimal projectors, the best correct visual acuity (BCVA) was assessed. Visual acuity was converted to LogMAR visual acuity.

### Operative details and sample handling

A single consultant vitreoretinal surgeon performed all PPV operations according to the same procedural guidelines. The same 23-gauge vitrectomy system (Combined Wide-Field Elite Pack, Bausch, and Lomb) was used for all operations. After administering anesthesia, at 4 mm from the limbus, three sclerotomies were created and three trocars were inserted. The first trocar was connected to the turned off infusion cannula. The infusion cannula was kept off to obain undiluated vitreous samples. The second and third trocars were connected to the endo-light and to the vitreous cutter, respectively. At a cut rate of 5000 cuts per minute (cpm), the vitreous cutter was utilized to obtain 1 to 2 mL of the mid vitreous in a 5 mL syringe attached to the cutter while keeping the infusion pump off. The vitreous samples were considered surgical by-products, and their collection did not alter or modify the surgical procedure, ensuring the patient’s safety and optimal surgical efficacy. No additional techniques were used to extract or collect the vitreous sample during the vitrectomy.

The obtained vitreous samples were labeled with the names of the participants, hospital identification numbers, and given assigned numbers and then transferred to sterile 2 mL Eppendorf tubes. Then, the samples were kept immediately at -80℃ until further analyses.

### Sample processing and biomarkers quantification

After being thawed, the vitreous samples were centrifuged at 20,000 *g* for 15 min at 4℃. The resulting supernatant was collected. The concentrations of VEGF and PEDF were determined using the enzyme-linked immunosorbent assay (ELISA) method. The assay was performed in duplicates following the manufacturer’s protocol (ABCAM, Cambridge, CB2 0AX, UK). The protocol involves the use of a monoclonal antibody that is specific for pre-coated biomarkers onto a microtiter plate. Quality control samples were used, and random analysis of previously quantified samples was conducted.

The ELISA test used to detect VEGF was able to identify two out of four VEGF isoforms, namely VEGF121 and VEGF165. These shorter isoforms are released while the two longer ones are cell-associated. To comply with the detection range of the relevant assay, 100-µL aliquots of vitreous samples were used, which were eventually diluted to contain the same amount of protein. For the VEGF ELISA, 100 µL of the standard solution was added to the wells of a 96-well plate coated with a monoclonal antibody. For PEDF ELISA, 50 µL of the standard solution was added. After process of incubation, the plate was washed followed by the addition of an enzyme-labeled antibody and the substrate. Once the color had developed, the reaction was ceased by adding the stop solution. The optical density of the ELISA plates was measured at 450 and 570 nm using an absorption spectrophotometer (Bio-Tek Instruments, Winooski, Vermont, USA). A standard curve was plotted using the measurements made with the standard solution (12.5 pg/mL − 800 pg/mL for VEGF, and 468.75 pg/mL − 30,000 pg/mL for PEDF). This curve was used to determine the concentration of VEGF and PEDF in each sample. The minimum detectable concentration was 2.7 pg/mL for VEGF (with an intra-assay coefficient of variation (CV) of 5.4%, and an inter-assay CV of 5.5%), and 146 pg/mL for PEDF (with an intra-assay CV of 2.1% and an inter-assay CV of 4.1%). The level of each factor in the vitreous was within the detection range of the relevant assay. The protein content was determined in aliquots of tissue specimens using the standard DC assay (BioRad Hercules, CA, USA).

### Statistical analysis

The collected data was inserted into a spreadsheet and analyzed statistically using the IBM SPSS statistical package for Windows v.26 (Armonk, New York, USA). Nominal variables were expressed as frequency (percentage) and continuous variables as mean ± standard error of the mean (SEM). The normality of the data was tested using the Kolmogorov-Smirnov test. The statistical significance between the study groups was determined by using the *Chi*-square test for categorical variables and the Student’s *t*-test for continuous variables. A statistically significant result was considered if *P* ≤ 0.05. Logistic regression analysis was employed to assess the correlation between the measured biomarkers and the prognostic indices.

## Results

### Clinical characteristics of the study cohort and differences between case and control groups

A summary of the clinical characteristics of the study cohort and the differences between the case and control groups is represented in Table 1. A total of 80 patients were enrolled in the study. The study participants were divided into case and control groups for comparative analysis (Table 1). The case group (*n* = 42) included patients with advanced PDR and the control group (*n* = 38) included patients with other diagnostic indications. The mean age of the study population was 52.5 years; the mean age of the case group (55.3 ± 1.4 years) was significantly higher than the control group (49.3 ± 2.7 years) with a *P* value of 0.046. More than half of the patients were males (56.3%). Gender distribution across the groups showed no statistical difference (*P* > 0.05); the case group comprised 47.6% male and 52.4% female, whereas the control group had 65.8% male and 34.2% female participants. Eye laterality was balanced with no significant difference between the groups (*P* > 0.05). DM and hypertension (HTN) were present in 52 of the patients. The number of patients with DM and HTN is significantly higher in the case group with 100% and 58.7% as compared to the control group with 26.3% and 42.1%, respectively with *P* values of 0.001 (Table 1).

**Table 1:** Demographical details of study population (*n* = 80) and comparison between case and control groups

Variables	Number (percentage) or mean ± SEM			
	Overall (*n* = 80)	Case group (*n* = 42)	Control group (*n* = 38)	*P*-value
**Age**	52.5 ± 1.5	55.3 ± 1.4	49.3 ± 2.7	0.046
**Gender**				
Male	45 (56.3)	20 (47.6)	25 (65.8)	NS
Female	35 (43.8)	22 (52.4)	13 (34.2)	
**Eye laterality**				
Right eye (OD)	42 (52.5)	20 (47.6)	22 (57.9)	NS
Left eye (OS)	38 (47.5)	22 (52.4)	16 (42.1)	
**Co-morbidities**				
Diabetes mellitus	52 (65.0)	42 (100)	10 (26.3)	0.001
Hypertension	52 (65.0)	36 (58.7)	16 (42.1)	0.001
**Treatment of DM**				
Oral hypoglycemic agents	20 (39.2)	15 (36.6)	5 (50)	NS
Oral hypoglycemic agents and/or insulin	31 (60.8)	26 (36.4)	5 (50)	
**Duration of DM (years)**	14.3 ± 1.1	15.1 ± 1.1	10.6 ± 2.8	NS
**Type of intravitreal injection**				
Aflibercept	12 (31.6)	10 (29.4)	2 (50)	NS
Ranibizumab	26 (28.4)	24 (70.6)	2 (50)	
**Number of intravitreal injections**	2.54 ± 0.4	4.2 ± 0.7	0.7 ± 0.4	0.00001
**Time elapsed since last intravitreal injection (days)**	26.93 ± 9.8	18.2 ± 8.2	91.8 ± 50.4	0.013
**Preoperative diagnosis**				
Advanced PDR (FVM and VH)	42 (52.5)	42 (100)	0 (0)	0.00001
Rhegmatogenous retinal detachment (RRD)	23 (28.7)	0 (0)	23 (60.5)	
Vitreomacular interface diseases (ERM/MH/VMT)	9 (11.3)	0 (0)	9 (23.7)	
Endophthalmitis	3 (3.8)	0 (0)	3 (7.9)	
Dropped IOL or crystalline lens	3 (3.8)	0 (0)	3 (7.9)	
**Associated cataract surgery with the primary PPV**				
Yes	39 (49.3)	30 (71.4)	11 (28.9)	0.0001
No	41 (50.7)	12 (28.6)	27 (71.1)	
**Type of vitreous tamponade**				
Silicone oil	38 (47.5)	15 (35.7)	23 (60.5)	0.035
Gas	22 (27.5)	12 (28.6)	10 (26.3)	
Air	20 (25)	15 (35.7)	5 (13.2)	
**Postoperative AG use**	25 (32.5)	11 (28.2)	14 (36.8)	NS
**Follow up period (months)**	17.2 ± 1.3	16.0 ± 2.1	17.8 ± 1.8	NS
**Visual outcomes (Log MAR)**				
Preoperative BCVA	1.40 ± 0.1	1.5 ± 0.1	1.3 ± 0.1	NS
BCVA after 1 month	0.97 ± 0.1	1.00 ± 0.1	0.9 ± 0.1	NS
BCVA after 3 months	1.03 ± 0.1	1.2 ± 0.1	0.9 ± 0.1	NS
BCVA at the last follow up	0.95 ± 0.1	1.0 ± 0.1	0.9 ± 0.1	NS
**HBA1c (%)**	8.21 ± 0.3	9.23 ± 0.3	6.06 ± 0.5	0.001
**Homocysteine (mcmol/L)**	18.53 ± 1.9	18.0 ± 2.0	19.22 ± 3.5	NS
**Vitreous biomarkers**				
VEGF (pg/mL)	3404.15 ± 520.3	5744.06 ± 761.5	817.94 ± 403.1	0.0001
VEGF/Protein (pg/mg)	2354.69 ± 331.3	4133.21 ± 488.2	388.95 ± 52.0	0.0001
PEDF (pg/mL)	4444.80 ± 956.2	3487.47 ± 710.2	5502.90 ± 923.0	0.046
PEDF/ Protein (pg/mg)	2398.10 ± 510.0	2873.08 ± 593.5	2924.80 ± 752.3	NS
Protein content (mg/mL)	1.76 ± 0.2	1.56 ± 0.1	2.00 ± 0.4	NS

**Abbreviations**: SEM: standard error; DM: diabetes mellitus; PDR: proliferative diabetic retinopathy; FVM: fibrovascular membrane; VH: vitreous hemorrhage; RRD: rhegmatogenous retinal detachment; MH: macular hole; VMT: vitreomacular traction; ERM: epiretinal membrane; IOL: intraocular lens; PPV: pars plana vitrectomy; AG: anti-glaucoma; BCVA: best corrected visual acuity; VEGF: vascular endothelial growth factor; PEDF: pigmented epithelial growth factor; NS: not significant

The number of administered intravitreal injections was significantly higher in the case group (4.2 ± 0.7) compared to the control group (0.7 ± 0.4), with a *P* value < 0.001. It is important to mention that only cases of ERM in the control group received intravitreal injections as part of the treatment protocol before the surgery. Preoperatively, all participants in the case group were diagnosed with advanced PDR (FVM or VH) as opposed to none in the control group. Participants in the control group were diagnosed with various conditions, including rhegmatogenous retinal detachment (60.5%), endophthalmitis (7.9%), dropped lens matter (7.9%), and VMT diseases (23.7%). The incidence of cataract surgery associated with primary PPV was significantly higher in the case group (71.4%) than in the control group (28.9%) (*P* = 0.001). The use of different types of retinal tamponade showed silicone oil was more significantly used in the control group (60.5%) than the case group (35.7%) (*P* = 0.035), while gas was equally used in both groups. However, air was utilized more significantly in the case group (Table 1).

The mean VEGF levels were significantly higher in the case group (5744.06 ± 761.5 pg/mL) than in the control group (817.94 ± 403.1 pg/mL) (*P* < 0.001). When normalized to protein content, VEGF/Protein concentration was also significantly different between the case (4133.21 ± 488.2 pg/mg) and the control (388.95 ± 52.0 pg/mg) groups (*P* < 0.001). Regarding the PEDF levels, on the other hand, an inverse trend was observed. The case group showed a significantly lower concentration of PEDF levels (3487.47 ± 710.2 pg/mL) compared to the control group (5502.90 ± 923.0 pg/mL) (*P* = 0.046). However, when accounting for protein content, the PEDF/Protein ratio did not significantly differ between case and control groups.

A comparative analysis was performed between the retinal pathology groups and clarified in (Table 2). It showed that the mean age of patients was significantly older in the VMT group. In addition, males were found to be more in the RRD group. PPV was associated more with cataract surgery in PDR group. Moreover, silicone was utilized more significantly in the RRD group, air was utilized more in the PDR group and gas was utilized more in the VMT group. Furthermore and as will be shown in the next section, VEGF level was much higher in endophthalmitis and PDR groups while PEDF was much higher in endophthalmitis, VMT, and RRD groups.

**Table 2:** A comparative analysis between all groups of retinal disease

Variables	Number (percentage) or mean ± SEM					
	Advanced PDR (*n* = 80)	RRD (*n* = 42)	VMT (*n* = 38)	Endophthalmitis	Dropped IOL/Lens	*P*-value
**Age**	55.3 ± 1.4	45.3 ± 3.1	62.3 ± 4.2 ↑	31.0 ± 9.3	59.0 ± 3.6	0.001
**Gender**						
Male	20 (47.6)	18 (78.3) ↑↑	5 (55.6)	0 (0.0)	2 (66.7)	0.04
Female	22 (52.4)	5 (21.7)	4 (44.4)	3 (100.0)	1 (33.3)	
**Associated cataract surgery with the primary PPV**						
Yes	29 (70.7) ↑↑	4 (17.4) ↓↓	5 (55.6)	2 (66.7)	0 (0.0)	0.001
No	12 (29.3)	19 (82.6)	4 (44.4)	1 (33.3)	3 (100.0)	
**Type of vitreous tamponade**						
Silicone oil	15 (35.7)	20 (87.0) ↑	0 (0.0)	2 (66.7)	1 (33.3)	0.001
Gas	12 (28.6)	3 (13.0) ↓	6 (66.7) ↑	1 (33.3)	0 (0.0)	
Air	15 (35.7) ↑	0 (0.0)	3 (33.3)	0 (0.0)	2 (66.7)	
**HBA1c (%)**	9.2 ± 1.2 ↑	6.5 ± 2.6	7.8 ± 2.8	5.4 ± 1.1	6.5 ± 1.3	0.001
**Homocysteine (mcmol/L)**						
**Vitreous biomarkers**						
VEGF (pg/mL)	5744.06 ± 760.1 ↑	418.16 ± 57.2	259.43 ± 46.1	6181.67 ± 90.7 ↑↑	194.67 ± 22.1	0.0001
PEDF (pg/mL)	3487.47 ± 730.8	4147.24 ± 101.1↑	5936.16 ± 283.1 ↑	18714.96 ± 8830.4 ↑↑	1384.49 ± 199.6	0.04

**Abbreviations**: SEM: standard error; PDR: proliferative diabetic retinopathy; RRD: rhegmatogenous retinal detachment; VMT: vitreomacular traction; IOL: intraocular lens; PPV: pars plana vitrectomy; VEGF: vascular endothelial growth factor; PEDF: pigmented epithelial growth factor

### Factors affecting the vitreous levels of VEGF and PEDF

Factors influencing the levels of VEGF and PEDF in the study cohort were evaluated (Table 3). The analysis revealed that several factors affected the levels of VEGF and PEDF within the patient cohort. For PEDF, age had a statistically significant negative association (-114.12 ± 56.2 pg/mL, *P* = 0.035), indicating lower PEDF levels with increasing age. Gender did not significantly influence PEDF levels, with males showing a mean of 4245.70 ± 384.8 pg/mL and females 4700.79 ± 539. 3 pg/mL. Co-morbid conditions such as DM, HTN, and chronic kidney disease did not significantly affect PEDF levels. Moreover, treatment and duration of DM did not significantly influence the PEDF levels. Preoperative diagnosis significantly influenced PEDF levels. Cases of endophthalmitis, VMT, and RRD were significantly associated with higher PEDF levels (18714.96 ± 8830.4, 5936.16 ± 283.1, 4147.24 ± 101.1 pg/mL, respectively, *P* = 0.04). The type of retinal tamponade was also a significant factor, with higher PEDF levels observed in patients with gas tamponade (6470.94 ± 567.1 pg/ml, *P* = 0.02). Multiple regression analysis revealed that endophthalmitis or RRD is the most important independent factor affecting PEDF levels.

**Table 3:** Factors affecting the level of VEGF and PEDF

Variables	Mean ± SEM* or B Regression Coefficient ± SEM **			
	VEGF (pg/mL)	*P*-value	PEDF (pg/mL)	*P*-value
**Age**	-18.1 ± 3.8	NS	-114.12 ± 56.2	0.035
**Gender**				
Male	2832.30 ± 674.9	NS	4245.70 ± 384.8	NS
Female	4139.40 ± 807.7		4700.79 ± 539. 3	
**Co-morbidities**				
Diabetes mellitus	4711.95 ± 681.5	0.00001	3796.67 ± 730.7	NS
Hypertension	3908.65 ± 647.4	NS	4499.06 ± 613.1	NS
Chronic kidney disease	5351.23 ± 1300.1	NS	1326.06 ± 593.0	NS
**Treatment of DM**				
Oral hypoglycemic agents	3769.47 ± 828.2	NS	3888.28 ± 516.2	NS
Oral hypoglycemic agents and/or insulin	5202.27 ± 1001.3		3818.86 ± 470.3	
**Duration of diabetes mellitus (years)**	165.30 ± 12.1	NS	33.98 ± 6.2	NS
**Number of intravitreal injections**	330 ± 13.0	0.013	-256.04 ± 11.6	NS
**Time elapsed since last intravitreal injection (days)**	-16.30 ± 1.3	NS	0.12 ± 0.6	NS
**Preoperative diagnosis**				
Advanced PDR (FVM and VH)	5744.06 ± 760.1 ↑	0.0001	3487.47 ± 730.8	0.04
Rhegmatogenous retinal detachment (RRD)	418.16 ± 57.2		4147.24 ± 101.1 ↑	
Vitreomacular interface diseases (ERM/MH/VMT)	259.43 ± 46.1		5936.16 ± 283.1 ↑	
Endophthalmitis	6181.67 ± 90.7 ↑↑		18714.96 ± 8830.4 ↑↑	
Dropped IOL or crystalline lens	194.67 ± 22.1		1384.49 ± 199.6	
**Associated cataract surgery with the primary PPV**				
Yes	4558.67 ± 456.9	0.028	4908.04 ± 846.6	NS
No	2261.05 ± 789.0		4046.91 ± 789.1	
**Type of vitreous tamponade**				
Silicone oil	2025.74 ± 520.3	0.036	4251.16 ± 643.9	0.02
Gas	4373.68 ± 1045.1		6470.94 ± 567.1 ↑	
Air	4956.66 ± 1391.6		2583.96 ± 989.5	
**Postoperative AG use**	2405.15 ± 567.4	NS	2508.78 ± 370.1	NS
**HBA1c (%)**	771.01 ± 29.8	0.013	351.66 ± 0.5	NS
**Homocysteine**	-10.36 ± 4.2	NS	10.47 ± 3.5	NS

**Abbreviations**: SEM: standard error; DM: diabetes mellitus; PDR: proliferative diabetic retinopathy; FVM: fibrovascular membrane; VH: vitreous hemorrhage; RRD: rhegmatogenous retinal detachment; MH: macular hole; VMT: vitreomacular traction; ERM: epiretinal membrane; IOL: intraocular lens; PPV: pars plana vitrectomy; AG: anti-glaucoma; VEGF: vascular endothelial growth factor; PEDF: pigmented epithelial growth factor; NS: not significant

Regarding the VEGF, age had a negative association with VEGF levels (-18.1 ± 3.8 pg/mL), though this was not statistically significant. Similarly, gender did not significantly influence VEGF levels. Co-morbid conditions such as DM were significantly associated with higher VEGF levels (4711.95 ± 681.5 pg/mL, *P* < 0.001). However, other co-morbidities like HTN and chronic kidney disease did not significantly affect VEGF levels. The time elapsed since the last intravitreal injection demonstrated a negative correlation with VEGF levels though not statistically significant (-16.0 ± 1.3 pg/mL, *P* > 0.05); thus as the duration increased by one day, the level of VEGF decreased by 16.0 pg/mL. Preoperative diagnoses significantly influenced VEGF levels. It was found that patients with endophthalmitis and advanced PDR (FVM and VH) had the highest levels of VEGF (5744.06 ± 760.1 pg/mL and 6181.67 ± 90.7 pg/ml, respectively, *P* < 0.001). Associated cataract surgery with the primary PPV also had a significant positive correlation with VEGF levels (4558.67 ± 456.9 pg/mL, *P* = 0.028). The type of retinal tamponade used post-PPV showed that silicone oil was associated with lower VEGF levels than gas and air (2025.74 ± 520.3 pg/mL for silicone oil, *P* = 0.036). The level of HbA1c significantly influenced the level of VEGF (771.01 ± 29.8 pg/mL, *P* = 0.013). Advanced PDR was revealed to be the most important independent factor affecting VEGF levels by multiple regression analysis.

### Factors affecting the level of PEDF within RRD cases

An analysis of 23 patients with RDD, mostly males (78.3%), was conducted to determine the factors affecting PEDF levels. The mean age of those patients was 45.3 years. Only four patients had DM, and eight patients had HTN. The mean HbA1c level for those diabetic patients was 6.50%. The right eye was involved in 14 of the cases. Regarding the macular status, the macula was attached (macula on) at the time of PPV in 7 cases (30.4%), and in the rest of the cases, the macula was detached (macula off). The tear was found superiorly in 16 (69.6%) cases and inferiorly in 7 (30.4%) cases. The mean duration of RRD symptoms was 6.28 days. Cataract surgery was associated with the primary PPV in four cases. Silicone oil was the primary tamponade used in 20 (87.0%) cases, while gas was used in three (13%) cases. The BCVA before surgery was 1.209 LogMAR. Post-surgery, the visual acuity was 0.774 LogMAR at three months and 0.895 LogMAR at the last follow-up visit.

Table 4 demonstrates the factors affecting PEDF levels in patients with RDD. The age of the patients was inversely proportional to the PEDF level (*P* = 0.047). The level of PEDF decreased by 227.37 ± 96.2 pg/mL for each year of age. Gender, co-morbidities, and the use of anti-glaucoma medications did not correlate with PEDF levels. However, the macular status was found to affect the level of PEDF. Patients with detached macula (macula off) had higher PEDF levels than patients with attached macula (5039.45 ± 770.3 pg/mL vs. 2107.88 ± 636.2 pg/mL, *P* = 0.046).

**Table 4:** Factors affecting the level of PEDF in patients with RRD

Variables	Mean ± SEM* or B Regression Coefficient ± SEM **	
	PEDF (pg/mL)	*P*-value
**Age**	-227.37 ± 96.2	0.047
**Gender**		
Male	3733.89 ± 684.1	NS
Female	5635.30 ± 939.4	
**Co-morbidities**		
Diabetes mellitus	1021.078 ± 364.7	NS
Hypertension	1179.89 ± 273.1	NS
Chronic kidney disease	1326.06 ± 593.0	NS
**Macula status**		
Macula on	2107.88 ± 636.2	0.046
Macula off	5039.45 ± 770.3	
**Duration of RRD (days)**	823.12 ± 124.2	0.001
**Location of the tear**		
Within the superior half	1794.99 ± 593.8	0.041
Within the inferior half	9523.79 ± 911.1	
**Associated cataract surgery with the primary PPV**		
Yes	2531.25 ± 846.6	NS
No	4487.44 ± 1011.7	
**Type of vitreous tamponade**		
Silicone oil	4167.12 ± 543.9	NS
Gas	2332.16 ± 467.1	
**Postoperative AG use**	2508.78 ± 370.1	NS
**HBA1c (%)**	-213.23 ± 56.1	NS
**Homocysteine**	-24.44 ± 12.5	NS

**Abbreviations**: SEM: standard error; RRD: rhegmatogenous retinal detachment; PPV: pars plana vitrectomy; AG: anti-glaucoma; PEDF: pigmented epithelial growth factor; NS: not significant, N=23

Moreover, in cases where the tear was found inferiorly, the level of PEDF was significantly higher than in patients with superior tears (9523.79 ± 911.1 pg/mL vs. 1794.99 ± 593.8 pg/mL, *P* < 0.05). Furthermore, the longer the duration of the detachment, the higher the level of PEDF. Furthermore, the level of PEDF increased by 823.12 ± 124.2 pg/mL for each day of detachment. The level of PEDF was not associated with the presence of cataract surgery, type of tamponade, or preoperative BCVA.

## Discussion

This is the most recent study investigating the factors affecting the levels of two main vitreous biomarkers, namely VEGF and PEDF. More importantly, this is the first study to investigate the relationship between the anatomical and clinical behaviour of RRD and the level of PEDF. It reveals that patients with advanced PDR have higher levels of VEGF. In addition, the levels of VEGF were higher with more intravitreal anti-VEGF injections and with increased levels of serum HbA1c. Patients diagnosed with endophthalmitis and advanced PDR showed higher levels of VEGF. PEDF was affected inversely by the age of the patients. Also, PEDF levels were higher in RRD, VMT, and endophthalmitis cases. Patients who had gas as retinal tamponade exhibited higher levels of PEDF. In patients with RRD, the level of PEDF was higher if the tear was found inferiorly and the macula was detached. Finally, the PEDF level was significantly higher with a longer duration of RRD.

During the detachment of the neurosensory retina from the RPE in the RRD, the ischemic changes trigger the expression of many growth factors and cytokines, such as VEGF, fibroblast growth factors, PEDF, and hepatocyte growth factor in the vitreous and subretinal fluid [20]. A study by Ogata et al. assessed the vitreous levels of PEDF in patients with RRD and advanced PDR and found that levels of PEDF were higher in cases of RRD than in cases of DR [9]. Zheng et al. revealed that vitreous PEDF levels were significantly lower in eyes with PDR than in eyes with idiopathic MH [21]. Dieudonné et al. studied the levels of PEDF by taking subretinal samples in scleral buckle surgery for RRD and vitreous samples for MH cases. They found that PEDF levels were substantially higher in RRD patients compared to MH patients [22]. In this study, the levels of VEGF were higher in patients with advanced PDR and the levels of PEDF were higher in patients with RRD. This could be explained by the angiogenic effects of VEGF and the anti-angiogenic effects of PEDF.

RPE is an important source for the production of PEDF in the eye and it was reported that deficiency of PEDF significantly increases ocular microvascular density [8]. Many experimental studies have revealed that vitreous levels of PEDF are significantly lower in patients with PDR [9, 23]. Additionally, elevated vitreous levels of VEGF are found in patients with PDR. Accordingly, it has been suggested that the imbalance between the vitreous levels of PEDF and VEGF may be the driven factor for the progression of DR and can shadow the lights on the possible role of PEDF as a protective factor in regulating anti-angiogenic homeostasis and neural activity. Zhang et al. demonstrated that in oxygen-induced retinopathy rats, PEDF significantly decreased by retinal capillary endothelial cells and Müller cells. In the current study, the PEDF levels in the vitreous of patients with RRD were higher than those of patients with advanced PDR. The high levels of PEDF in the vitreous may be derived from the interphotoreceptor matrix through the retinal tear during the RRD process. Since PEDF is a neuro-protective factor [24], the high levels of PEDF in eyes with RRD suggest it may carry out a neuroprotective function for the detached retina. Another observation that supports this theory is the finding of a previous study that revealed patients who received retinal photocoagulation had higher levels of PEDF and low levels of VEGF [25]. Our study disclosed new findings in that the levels of PEDF were higher if the retinal tear was found inferiorly in RRD. Also, it was higher if the macula was detached and if the duration of the detachment was longer. These factors may potentiate the release of more PEDF since inferior tears, macular detachment, and longer duration may result in more subretinal fluid and retinal damage [26].

Increased levels of VEGF can lead to abnormal changes in retinal vasculature, while anti-VEGF treatment prevents intraocular neovascularization and recovers visual function [27]. The vitreous levels of VEGF have been linked to PPV, which is a surgical treatment for advanced PDR, as a prognostic factor that can predict the outcome and complications of PPV, such as early postoperative VH. Nevertheless, the pathologic neovascularization and proliferative changes may continue to progress after PPV surgery [28]. Many investigators revealed that the levels of VEGF were significantly reduced in the vitreous of patients with advanced PDR after successful PPV [29]. However, a high VEGF level may still be maintained in the vitreous cavity after PPV, suggesting that PPV cannot stop the secretion of VEGF in the vitreous cavity [30]. Our results support the evidence that the levels of VEGF were much higher in the advanced PDR group than in the control group. This is attributed mainly to the ischemic insults by DR.

## Conclusions

This study sheds the light on important aspects of the vitreoretinal surgery. VEGF is the most important biomarker in PDR. Medications agains VEGF are the mainstay of the treatment of DR. Promising treatment are arising continuesly. PEDF is another important biomarker which may affect and predict the outcome of cases of RRD. More studies and trials are needed to justify these results and make a basis for therapeutic regimens and prognostic outcomes.

## Data Availability

No datasets were generated or analysed during the current study.

## Abbreviations

PEDF: Pigment epithelium-derived factor
VGEF: Vascular endothelial growth factor
PPV: Pars plana vitrectomy
DM: Diabetis mellitus
PDR: Proliferative diabetic retinopathy
ERM: Epiretinal membrane
RRD: Rheugmatogenous retinal detachment
TRD: Tractional retinal detachment
FVM: Fibrovascular membrane
DR: Diabetic retinopathy
VH: Vitrous hemorrhage
MH: Macular hole
VMT: Vitreomacular traction: BCVA: best corrected visual acuity
ELISA: Enzyme-linked immunosorbent assay
CV: Coefficient of variation
HTN: Hypertension
